# Phytochemical Profile and Antioxidant Activity of Sesame Seed (*Sesamum indicum*) By-Products for Stability and Shelf Life Improvement of Refined Olive Oil

**DOI:** 10.3390/antiox11020338

**Published:** 2022-02-09

**Authors:** Mohamed K. Morsy, Rokayya Sami, Eman Algarni, Amina A. M. Al-Mushhin, Nada Benajiba, Almasoudi A., Abeer G. Almasoudi, Enas Mekawi

**Affiliations:** 1 Department of Food Technology, Faculty of Agriculture, Benha University, Benha 13736, Qaluobia, Egypt; 2 Department of Food Science and Nutrition, College of Sciences, Taif University, P.O. Box 11099, Taif 21944, Saudi Arabia; eman1400@tu.edu.sa; 3 Department of Biology, College of Science and Humanities in Al-Kharj, Prince Sattam Bin Abdulaziz University, Al-Kharj 11942, Saudi Arabia; a.almushhin@psau.edu.sa; 4 Department of Basic Health Sciences, Deanship of Preparatory Year, Princess Nourah bint Abdulrahman University, P.O. Box 84428, Riyadh 11671, Saudi Arabia; nabenajiba@pnu.edu.sa; 5 Chemistry Department, Faculty of Science, King Abdulaziz University, P.O. Box. 42734, Jeddah 21551, Saudi Arabia; asalmasoudi@kau.edu.sa; 6 Food Science Department, College of Science, Branch of the College at Turbah, Taif University, Taif 21944, Saudi Arabia; a.sleman@tu.edu.sa; 7 Department of Agricultural Biochemistry, Faculty of Agriculture, Benha University, Benha 13736, Qaluobia, Egypt; enas.ibrahim@fagr.bu.edu.eg

**Keywords:** refined olive oil, sesame by-product, stability, natural antioxidants, phytochemical

## Abstract

The by-product of sesame seed coats from the tahini industry was used for the extraction of bioactive compounds as novel antioxidants. This study was designed to evaluate the effect of a natural antioxidant on the quality of refined olive oil (ROO) stored at 60 ± 1 °C for up to 48 days. The lyophilized sesame seed coats extract (LSSCE) was placed into fresh ROO at three levels, i.e., 200, 400, and 600 mg kg^−1^, and compared with 200 mg kg^−1^ BHT (reference) and without antioxidant (control). LSSCE exhibited high phenolic (105.9 mg GAE g^−1^) and lignin (6.3 mg g^−1^) contents as well as antioxidant activity based on HPLC/DAD. In ROO samples, Including LSSCE, the values of peroxide, *p*-anisidine, K_232_, and K_270_ were remarkably lower than control during storage. The kinetic rate constant (k) of oxidation indicators was the lowest in ROO samples containing BHT and LSSCE 600 mg kg^−1^compared with other treatments. LSSCE improved the organoleptic acceptability of ROO samples up to 48 days of storage. Moreover, the shelf life (assuming a Q_10_ value of 2.0 for lipid oxidation) of ROO treated with LSSCE was increased. The findings revealed that LSSCE is a promising natural antioxidant in delaying oxidation, enhancing oil stability, and prolonging the shelf life (~475 days at ambient temperature).

## 1. Introduction

In recent years, extensive research has been carried out on the potential of food by-products as an antioxidant-rich source that could be applied as food additives [1,2]. Bioactive compounds extracted from byproducts (agro-food chain) could be one of the promising ways to produce natural antioxidants [3], including phenolics, flavonoids, carotenoids, saponins, and alkaloids [4].

Artificial antioxidants, such as tertiary butylated hydroquinone (TBHQ), butylated hydroxytoluene (BHT), and butylated hydroxy anisole (BHA), have been successfully applied to inhibit oil oxidation for 50 years; however, they have health risks, such as toxic and cancer-related compounds [5]. Thus, the use of synthetic antioxidants in the food industry is progressively restricted [6]. Natural antioxidants, particularly those derived from food by-products, have recently received attention, such as potato peels [7], tomato pomace [3], pomegranate husks [8], cocoa by-product [9], and olive by-product [10]. Furthermore, natural antioxidants have a plethora of advantages, such as being healthy, safe, and inexpensive.

Sesame (*Sesamum indicum* L.) is a major oilseed crop grown in tropical regions, primarily Asia and Africa [11]. It is considered one of the oldest crops grown in Egypt (~1500 BC) [12]. According to FAO statistics, the global production of sesame seeds is approximately 5.53 million metric tons, with Egypt producing approximately 40,000 tons in 2017 [13]. In Egypt, sesame is industrially processed to obtain different products, such as sesame oil, tahini, and tahini halva [14]. These products are widely consumed in the Middle East, Mediterranean, North Africa, USA, and Europe [15]. During the processing of tahini and/or halva, the sesame seeds need to deshell and then produce a by-product called seed coats.

Sesame seed coats (SSCs) are by-products of tahini manufacturing or oil processing, including testae, bran, and hull [16]. The SSCs represent about 12% of the whole seed [14]. The SSCs are cheap, available, and generated in a large quantity [17]. Moreover, they are rich in bioactive compounds, such as polyphenolic sesamin and sesamolin [18]. The SSCs have remarkable antioxidants with high efficiency for inhibiting LDL oxidation and free radical scavenging [19]. Additionally, the phenolics have many health advantages, i.e., anti-inflammatory and anticancer [20]. The antioxidant impact of SSCs has been investigated in sunflower oil [14] and in macrophages [19]. One study by Mehdi et al. [21] reported that the oxidative stability of sesame oil obtained from whole seeds was higher than those extracted from de-husked seeds. Another study by El-Roby, Hammad, and Galal [14] found that ethanolic extract of sesame seed coat has an antioxidant activity similar to tocopherol. Prakash et al. [22] demonstrated that a black sesame coat had a higher antioxidant activity compared to a white sesame coat.

Refined olive oil (ROO) is obtained from virgin oils during the refining step without altering the initial glyceride structure, in accordance with Egyptian Standards 49-2/2005 [23] and Codex Standard [24]. ROO is widely consumed in Egypt and around the world because it is a reliable source of monounsaturated fatty acids (MUFA), which have health properties [25]. However, ROO has a short shelf life due to the partial removal of phenols during the refining process [26]. Consequently, ROO becomes more sensitive to oxidative stress and loses quality, requiring the addition of antioxidants to improve acceptability and extend shelf life [27]. Few studies have focused on natural antioxidants-ability to improve ROO stability, such as spirulina extract [28], tomato peels [3,29], and olive leaf extract [30]. Thus far, no research has been carried out on the use of sesame seed extract (SSCE) as a novel antioxidant to increase the stability of refined olive oil. Therefore, the aim of this study was, on the one hand, to assess the antioxidant capacity of lyophilized sesame coats extract (LSSCE) in comparison with butylated hydroxytoluene (BHT), and on the other, to assess the influence of LSSCE on the stability of ROO at high temperatures up to 48 days.

## 2. Materials and Methods

### 2.1. Chemicals and Raw Materials

Chemicals, i.e., gallic acid, ascorbic acid, acetic acid, sodium thiosulphate, sodium hydroxide, potassium iodide, starch, ethanol 95%, methanol, hexane, butanol, iso-octane, panisidine, Folin-Ciocalteu’s phenol reagent, butylated hydroxytoluene (BHT), 2, 2-diphenyl-1-picrylhydrazyl (DPPH), sesamol, sesamin, and sesamolin, were supplied from Sigma-Aldrich Company (St. Louis, MO, USA) and Merck Company (Darmstadt, Germany). All chemicals were of HPLC grade. Refined olive oil (ROO) without antioxidants, season 2020, was freshly supplied from Khoshala Olive Oil Company (Obour city, Cairo, Egypt). The samples were directly transported to the oil technology lab, Biochemistry Department, Benha University, and placed in a dark condition at ambient temperature. Dark sesame seed coats (SSCs) by-products were obtained from El-Bawady Company (Alexandria, Egypt). The powdered SSCs was packed and stored in a dry and dark environment until preparation.

### 2.2. Lyophilized Sesame Seed Coats Extract (LSSCE)

The lyophilized sesame seed coats extract (LSSCE) was prepared as described by Tabaraki et al. [31]. The sesame seed coats (SSCs) samples were ground in an electric coffee grinder (Braun Aromatic Coffee Grinder KSM2) to a fine powder and sieved by a 30-mesh sieve. The bioactive compounds of SSCs were extracted by ultrasonic bath (Bandelin Super Sonorex RK-100H, Altenberge, Germany). Ten grams of SSCs sample and 200 mL of aqueous ethanol (90%) were placed into a conical flask and sonicated for one hour at 25 °C. The sesame seed coats extract (SSCE) was filtered using Whatman No. 1 and concentrated under vacuum using a rotary evaporator (Laborota 4000, Heidolph, Germany) at 65 ± 1 °C, 100× *g* for 15–30 min. The SSCE were immediately frozen (–40 ± 1 °C), then dehydrated (20 ± 1 °C) for 50 h in a lyophilized device (Labconco 74200, Kansas City, KS, USA) under a 0.12 mbar and condensed (–85 ± 1 °C). The lyophilized sesame seed coats extract (LSSCE) was packed under vacuum polyethylene pouches and kept at –20 ± 1 °C. 

### 2.3. Refined Olive Oil and LSSCE

The fresh refined olive oil (ROO) was split into five groups based on LSSCE addition. The LSSCE were put into ROO samples in three groups at levels of 200, 400, and 600 mg kg^−1^. The fourth group contained BHT at 200 mg kg^−1^ as reference treatment, and the fifth group was free from antioxidants (control). The antioxidants were dissolved gently in ROO at 45 ± 1 °C for 20 min. All treatments were packed in a brown glass bottle and kept in an incubator under accelerated oxidation conditions (60 ± 1 °C for 48 days). The stability of oil samples was checked at intervals of 0, 6, 12, 18, 24, 30, 36, 42, and 48 days.

### 2.4. Phenolic and Flavonoid Contents of LSSCE

Total phenolic (TP) of LSSCE was measured using the Folin-Ciocalteu method at 750 nm, in accordance with Hajimahmoodi et al. [32]. Briefly, 200 µL of LSSCE (1 mg/mL) were added to 3 mL distilled water (DW), mixed gently with Folin-Ciocalteu reagent (0.5 mL for 3 min), followed by the addition of 2 mL sodium carbonate (20%; *w/v*). The mixture was left in the dark for 60 min, and absorbance was measured at 750 nm. TP was exposed as a mg of gallic acid equivalent (GAE) per 100 g of SSCs. Total flavonoid (TF) of LSSCE was determined colorimetric at 510 nm as described by Formagio et al. [33]. In brief, 50 µL of LSSCE (1 mg/mL) was added to 1 mL methanol, 4 mL of DW was added, 0.3 mL of NaNO_2_ (5%; *w/v*) and 0.3 mL of AI CI 3 (10%; *w/v*) was added after 5 min of incubation, and the mixture was left for 6 min. A total of 2 mL of NaOH (1 mol/L) was added. The final volume of the mixture was brought to 10 mL with double-DW. The mixture was left for 15 min, and absorbance was measured at 510 nm. TF was computed as mg rutin equivalents (RE) per gram of LSSCE. The analysis was performed in triplicate.

### 2.5. Antioxidant Ability of LSSCE

The DPPH radical scavenging ability of LSSCE was tested using the method reported by Liu et al. [34]. Briefly, 200 µL of LSSCE was mixed with 3.8 mL DPPH solution and incubated at ambient temperature in the dark for 60 min. The absorbance of the mixture was measured at 517 nm. The BHT (positive control) was also run. Data were exposed as IC_50_ (µg mL^−1^) using standardized material (ascorbic acid) as in [35].

### 2.6. HPLC-DAD of LSSCE 

#### 2.6.1. Determination of Phenolics 

The HPL Canalysis was performed by Agilent Technologies-1100 series chromatography (Santa Clara, CA, USA) [36], which included a self-sampler and a detector (DAD). The HPLC column was Agilent Eclipse XDB-C_18_, ID 4.6 mm, length 150 mm, and particle size, 5 μm (4.6 × 150 mm, 5 μm) with a C_18_ guard column. The mobile phase was two solvents: solvent (1) was acetonitrile and solvent (2) was acetic acid in acetonitrile (0.5%: 99.5%; *v/v*). The elution was by gradient elution starting with 100% of solvent (1) and ending with 100% of solvent (2). A DAD detector wavelength of 254 nm was used. The flowrate was run at 0.8 mL min^−1^ with a total time of 70 min. All samples were filtered (0.45 µm) with an Acrodisc syringe filter (Gelman Laboratory, Ann Arbor, MI, USA). The dose volume was 10 µL. Peaks were identified by consistent retention time and UV spectra and compared with standards.

#### 2.6.2. Determinations of Lignins (Sesamin and Sesamolin)

Lignins (sesamin and sesamolin) were extracted according to Chang et al. [37]. The LSSCE were mixed with methanol (1:10; *w/v*) and stirred. The mixture was centrifuged (Thermo Fisher Scientific, Newport, UK) at 10,000×*g* for 15 min under cool and the supernatant was kept at 4 °C. The lignins have been accomplished using the HPLC Agilent Technologies-1100 series. Water (phase 1) and methanol (phase 2) made up the mobile phase. The gradient solvent was increase of 10–90% of methanol from 0–70 min with a post-time of 5 min at a flow rate of 0.8 mL min^−1^. The dose volume was 10 μL and chromatograms were performed at 280 nm. Compared with previous reports and standards, a single lignan compound was identified based on the DAD spectrum and retention time [38]. A sesamin standard curve with a range of 0–1000 mg L^−1^ was prepared for the quantification of lignan compounds. The data were displayed as mg of sesamin meq kg^−1^of sesame coats (DM) [20].

### 2.7. Fatty Acids Profile

Fatty acids methyl esters (FAMEs) of ROO were obtained according to Ackman [39]. Briefly, 25 mg of ROO was gently mixed with 1.5 mL of NaOH in 0.50 mol L^−1^ methanol, and the mixture was heated (bath; ~100 °C; 5 min), then cooled down to ambient temperature. The mixture and 2.0 mL BF3 (12%) in methanol were mixed and heated again (bath; ~100 °C; 30 min), 1 mL of isooctane was added, and afterward, cooled to ambient temperature. It was stirred for 30 s before adding 5 mL of saturated NaCl. The esterified sample was kept in a refrigerator and allowed to rest for better phase separation. After collecting the supernatant, 1 mL of isooctane was added to the tube and stirred again. The supernatant and the previous fraction were mixed. The sample was concentrated in a final volume (1 mL) for later injection into GC. The analysis of FAMEs was performed by GC-FID (Perkin Elmer, Clarus 500 series, Midland, ON, Canada) under the following condition: Column, HP Innowax capillary: 60.0 mm × 0.25 mm × 0.25 µm. The heating temperature was 160 °C/20 min^−1^, then programmed at 10 °C min^−1^ until 240 °C. The temperature of the injector and detector was 220 and 250 °C, respectively. The carrier was hydrogen gas, flowrate 2.4 mL min^−1^, split ratio 40:1, and injector volume 1 μL. Peaks were identified by comparing the retention time of pure FAME standards (Larodan, Sweden).

### 2.8. Quality Parameters of Olive Oil and Chemical Kinetic

The peroxide value (PV) of ROO treatments was measured according to AOCS [40]. The results were exposed as meq kg^−1^ of oil. The *p*-Anisidine value (AV) of ROO was tested using the UV-Vis spectrophotometer (CE 599 Universal, Allentown, PA, USA) at 350 nm according to Paquot and Hautfenne [41]. The total oxidation (Totox) value was calculated by the following Equation (1):

Total oxidation (Totox) = 2 × PV + AV (1)

where PV is the peroxide value and AV is the *p*-Anisidine value.

The conjugated dienes (K_232_) and conjugated trienes (K_270_) were measured by spectrophotometry according to AOCS [40]. The kinetic (*k*) constants of zero-order reactions were assumed from the line slopes measured by the plotting concentration and time Gómez-Alonso et al. [42]. The temperature acceleration factor (Q10) was calculated from the line slopes and depended on the raise in oxidation level per 10 °C raise in temperature according to Steele [43] from the Equation (2):

Q_10_ = e^10 b^ (2)

where e is a constant and b is the Δ temperature.

### 2.9. Antioxidant Indices

An induction period (IP) of ROO was measured by the rancimat (Metrohn 679 AG, Herisau, Switzerland) [44]. The test condition was 100 °C and an airflow rate of 20 L h^−1^ in a reaction tube. The findings were displayed as induction time. Antioxidant power (AOP) and improved oxidative stability (IOS) were evaluated as outlined by the International Olive oil Council [45]. The results were computed using Equations (3) and (4):

(3) $$\mathrm{Antioxidant}\;\mathrm{power}\;(\mathrm{AOP})=100-\left[\left(\frac{IPcontrol}{IPsample}\right)\times 100\right]$$

(4) $$\mathrm{Improved}\;\mathrm{oxidative}\;\mathrm{stability}\;(\mathrm{IOS},\;\%)=\left(\frac{IPsample-IPcontrol}{IPsample}\times 100\right)$$

### 2.10. Organoleptic Assessment 

Assessment was performed by a 10-trained member panel (aged 20–45 years) experienced in organoleptic evaluation of olive oil from Biochemistry and Food Technology Departments. The assessment of ROO was in accordance with the IOC [46]. The olive oil treatments were put into brown glasses approximately 25 mL and code-3-random numbers. The panelists were judged to assess all oil samples using hedonic scale method (seven-point) for color, aroma, and acceptability, where 7 signifies excellent and 1 signifies unacceptable [47].

### 2.11. Data Analysis

The experiments were carried out in three replications for the antioxidant capacity, on three factors with three concentrations: (LSSCE, BHT, and replication). For ROO tests, i.e., PV, AV, totox, K_232_, K_270_, and organoleptic assessment, factorial design ANOVA with two factors with five concentrations (control, BHT, LSSCE 200 mg kg^−1^, LSSCE 400 mg kg^−1^, and LSSCE 600 mg kg^−1^) and storage time with nine points (0, 6, 12, 18, 24, 30, 36, 42, and 48 days) were performed for each parameter using SPSS (version 19; Chicago, IL, USA). Mean values at (*p <* 0.05) were conducted by Tukey’s test [48].

## 3. Results and Discussion

### 3.1. ROO Profile and Phenolic Compounds

Table 1 presents the composition analysis of ROO fatty acids. The results demonstrated that ten compounds were identified in ROO, while the main fatty acids in ROO were oleic acid (73.35%), palmitic acid (12.32%), and linoleic acid (9.12%). Additionally, ROO has a high content of monounsaturated fatty acids (MUFAs), which was about 74.18%; however, it contains few polyunsaturated fatty acids (PUFAs), i.e., 9.73%. The obtained results are in agreement with the International Olive Oil Council Standard [45] and also in agreement with those reports that showed that ROO was rich in MUFAs and oleic acid [28,49]. Moreover, ROO also contains phytochemicals, such as 0.281 mg GAE g^−1^ of phenolic compounds (data not displayed). One study by Alavi and Golmakani [49] showed that TPC in ROO was 0.294 mg GAE g^−1^, while another study by Morsy et al. [28] found that TPC in ROO was 0.266 mg GAE g^−1^. The slight changes in the TPC value of ROO might be due to the sesame variety and/or refined processes. Furthermore, Saadet al. [50] and Genovese et al. [51] demonstrated that major phenolics in olive oil are tyrosol, hydroxytyrosol, and oleuropein.

### 3.2. Phytochemicals and Antioxidant Activity of Lyophilized Sesame Seed Coats Extract (LSSCE)

As shown in Table 2, the phytochemicals in sesame seed coats, suchas total phenolic (TP) and total flavonoid (TF) compounds, were evaluated. The results demonstrated that the total phenolic (TPC) content in sesame seed coats (SSCs) and lyophilized sesame seed coats extract (LSSCE) was 85.12 and 105.9 mg GAE g^−1^ (DM), respectively. Shahid iet al. [52] found that the phenolic content of sesame hull was 29.7 mg GAE g^−1^, while Sarkis et al. [53] reported that the total phenolic content in sesame seed cake was 30.04 mg GAE g^−1^. The variation in TP might be due to the sesame variety or the extraction technique. The TF in SSCs and LSSCE was 7.45 and 9 mg GAE g^−1^, respectively. The data were consistent with the findings by El-Roby et al. [14]. Additionally, Table 2 exhibits the antioxidant activity of sesame coats (DPPH). The radical scavenging capacity of SSCs and LSSCE was similar to that of α-tocopherol; however, it was lower than BHT’s. A study by Chang et al. [37] found that sesame coats have a 94.9% scavenging impact on DPPH and marked reducing power. Therefore, our results confirmed that LSSCE had a stronger antioxidant capacity than α-tocopherol, but weaker than BHT. The findings imply that LSSCE can be utilized instead of antioxidants to in oil or fatty foods.

### 3.3. HPLC Fingerprint of LSSCE

**Figure 1:**
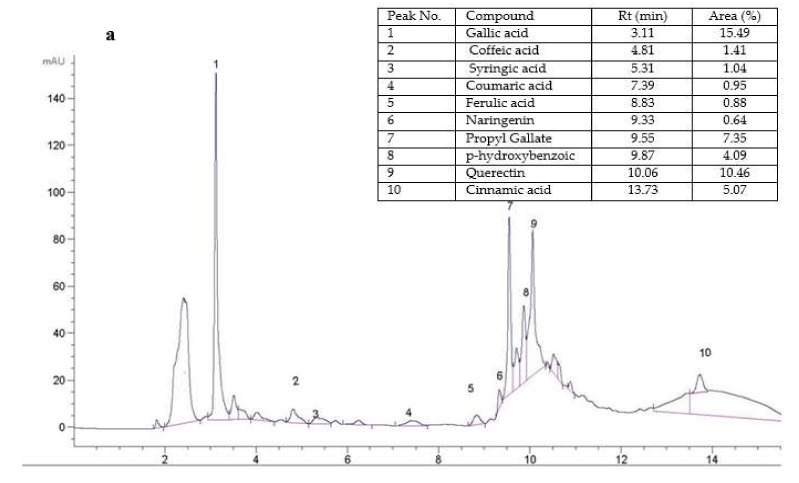

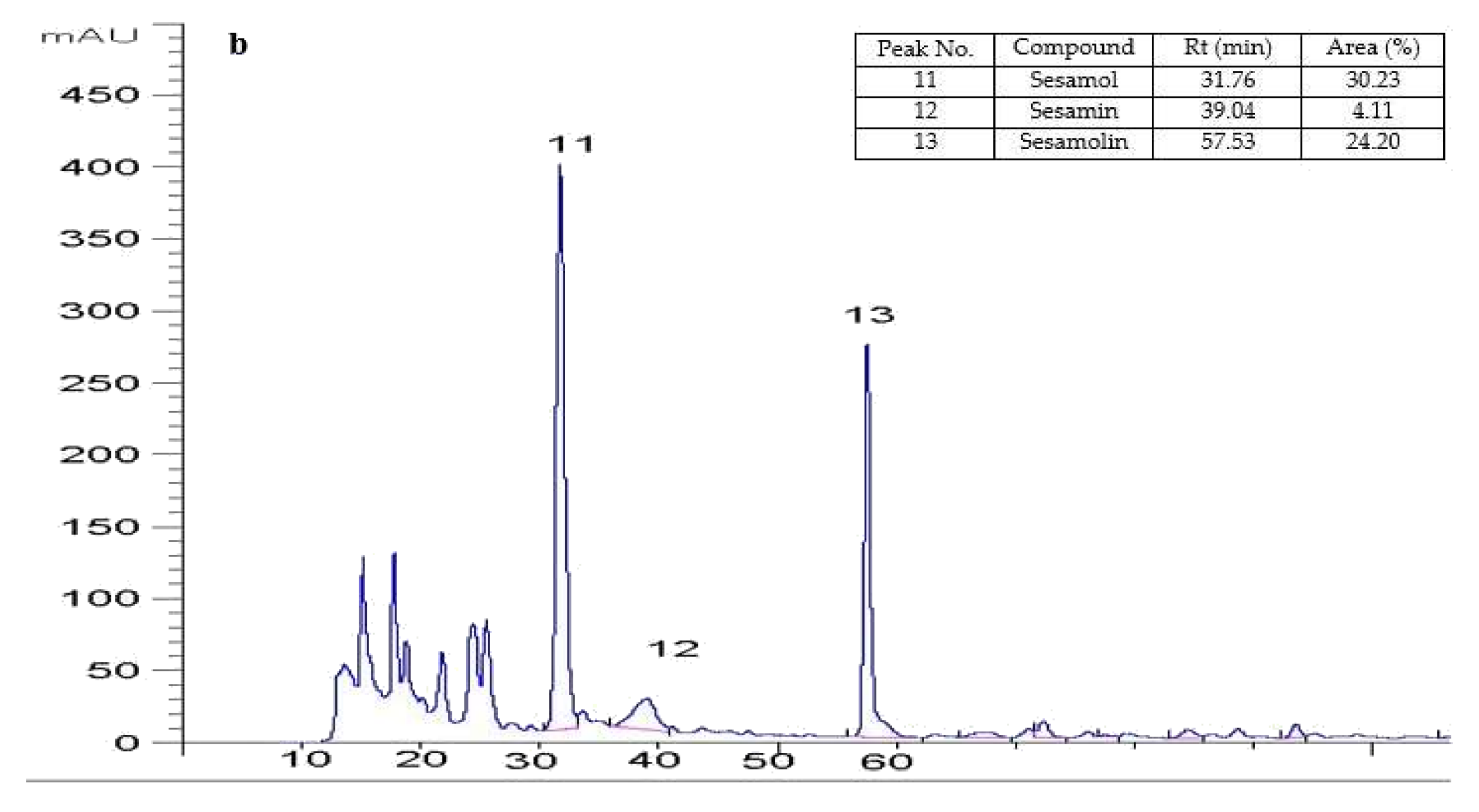
(**a**) HPLC fingerprints of LSSCE were identified as gallic acid (peak 1), coffeic acid (peak 2), syringic acid (peak 3), coumaric acid (peak 4), ferulic acid (peak 5), naringenin (peak 6), propyl gallate (peak 7), p-hydroxybenzoic (peak 8), querectin (peak 9), and cinnamic acid (peak 10); (**b**) HPLC fingerprints of lignins in LSSCE were identified as sesamol, (peak 11), sesamin (peak 12), and sesamolin (peak 13).

The phenolic and lignan compounds were determined and presented in Figure 1a. Results confirms that in LSSCE, there is a high concentration of total phenolic and lignan compounds. The HPLC identified the major phenolic and lignan compounds (~13 peaks) in LSSCE. Ten components of phenolic were recognized in LSSCE, having the following order: gallic acid >querectin> propyl gallate > cinnamic acid > p-hydroxybenzoic > caffeic acid > syringic acid > coumaric acid > ferulic acid > naringenin. Gallic acid represented 15.49% of the ten phenols and was the most important component. These findings are consistent with those reported by Elhanafi et al. [54], who found that gallic acid was the most important phenolic compound in sesame coats. However, El-Roby et al. [14] showed that coumaric acid and catechin were the most important phenolic compound in sesame coats. The presence of specific phenolic compounds may be influenced by different sesame varieties as well as other factors, i.e., soil fertility and sunlight [55].

The lignins, such as sesamol, sesamin, and sesamolin, were identified in LSSCE Figure 1b. The results revealed that the contents of sesamol, sesamin, and sesamolin were 3.30, 0.44, and 2.56 mg g^−1^, respectively. The sesamin: sesamolin ratio was 1.25, whereas Wang et al. [56] showed that the sesamin: sesamolin ratio ranged from 1.28 to 1.82 in sesame. Shiet al. [57] measured a sesamin level between 1.11 and 9.41 mg g^−1^ seed, and a sesamolin level between 0.20 and 3.35 mg g^−1^ seed.

### 3.4. Effect of LSSCE on Stability of ROO

**Figure 2:**
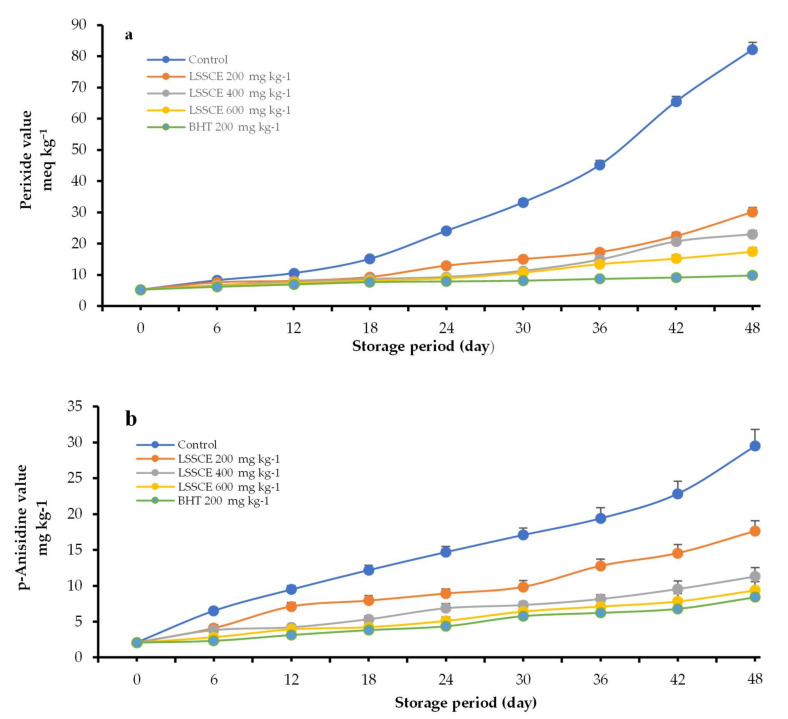

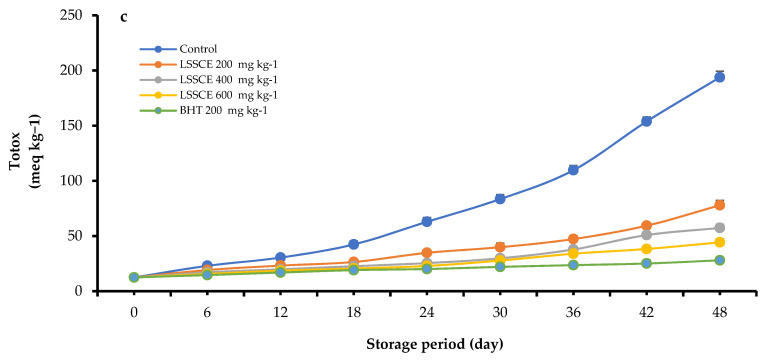
Impact of LSSCE on peroxide value (**a**), p-anisidine value (**b**), and totox value (**c**) in ROO stored at 60 °C.

The stability of ROO is usually determined using accelerated oxidation storage at temperatures >50 °C. Parameters such as peroxide (PV), *p*-Anisidine (AV), totox (TV), K_232_, and K_270_ values are among those tested for the assessment of rancidity in oils, owing to the formation of peroxides and hydroperoxides during the initial stages of oil oxidation [28,58]. All prior parameters were assessed in this experiment to track the primary and secondary oxidation status ROO samples during 48 days at 60 °C Figure 2.

#### 3.4.1. Peroxide Value (PV)

As shown in Figure 2a, the impact of antioxidants and storage condition on PV was evaluated and compared to the control group. The results demonstrated that ROO samples with added antioxidants, i.e., LSSCE and/or BHT, were more stable during the storage time when compared to the control sample. The PV of all treated ROO samples increased during the storage period, but with varying levels. During the first 12 days, the PV variation of ROO was slow, which was linked to the induction phase of oil oxidation. After this period, the PV values increased gradually, but to a higher extent for the control sample. The ROO enriched with LSSCE extracts was more resistant to oxidation compared to the control sample at all intervals. This could be due to the protective impact of bioactive compounds, such as the phenolic compounds present in LSSCE, on hydroperoxides formation and reduction of the oil oxidation, consistent with previous studies [14,54]. In the control sample, the PV had reached the maximum limit (>20 meq O_2_ kg^−1^ oil) on day 24, followed by a steady increase of 82.16 meq O_2_ kg^−1^ at the end of self-oxidation. However, the PV in oil samples incorporating antioxidants such as BHT, LSSCE 600 mg kg^−1^, LSSCE 400 mg kg^−1^, and LSSCE 200 mg kg^−1^ were less than the limiting value until day 48, 48, 36, and 36, respectively. Moreover, the PV of the ROO treated with BHT was remarkably lower than LSSCE [14].

#### 3.4.2. p-Anisidine Value (AV) and Totox Value (TV)

Figure 2b showed that antioxidant addition and keep condition had a significant impact (*p* ≤ 0.05) on the AV. The AV refer to conjugation compounds in oil, mostly 2-alkenes [59]. A progressive increase in the AV was observed in the control sample from 2.07 to 12.17 mg kg−^1^ until day 18 of storage, while at the end of the oxidation stage, it increased further to 29.48 mg kg^−1^. However, the treated samples ROO with antioxidants such as BHT or LSSCE were more stable during storage. The threshold/limit of the AV in ROO is <10 mg kg^−1^ [60]. The addition of LSSCE 600 mg kg^−1^enhanced the stability of ROO up to 48 days (<10 mg kg^−1^) in parallel with TBH. This protective effect of LSSCE could be due to the phenolic compounds. The findings have been matched with those reported by Changet al. [37], who found that polyphenolic compounds in sesame coats are associated with antioxidant capacity and have an important role in stabilizing lipid oxidation.

Similarly, a concomitant increase in totox value (TV) (Figure 2c) was observed for all the samples during 48 days of storage time. The totox value of ROO samples that included LSSCE at different levels was lower than the control. Therefore, the TV had following order: BHT < LSSCE 600 mg kg^−1^ ˂ LSSCE 400 mg kg^−1^ ˂ LSSCE 200 mg kg^−1^. These results are in agreement with those reported by Zhouet al. [61].

#### 3.4.3. K_232_ and K_270_ Values

**Figure 3:**
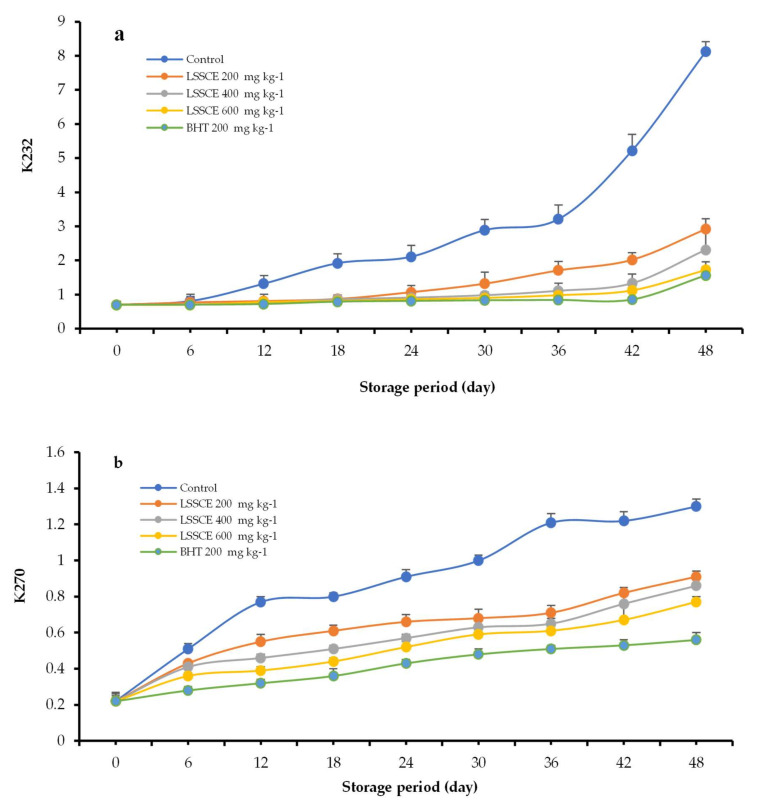
Impact of LSSCE on K_232_ (**a**) and K_270_ (**b**) in ROO stored at 60 °C.

The K_232_ and K_270_ values are indicators of primary and secondary oxidation in ROO, respectively [28]. Significant differences (*p* ≤ 0.05) in the K_232_ values of different ROO samples incorporating antioxidants were measured compared to the control sample (Figure 3a). The K_232_ values of all treated ROO gradually increased during storage. The ROO samples that included LSSCE had a notably lower than those of the control group, while the sample containing BHT had a K_232_ value of 0.56, which is a lower value than those of the LSSCE at differ levels. The K_232_ threshold/limit of ROO is ˂2.6. Additionally, the K_270_ increased rapidly (*p* ≤ 0.05) in the control group compared to treated ROO samples (including natural or artificial antioxidants) (Figure 3b). The K_270_ threshold/limit of ROO is ˂0.9. The results demonstrated that the addition of LSSCE at 400 and 600 mg kg^−1^ in ROO ensured that the K_270_ value was less than the threshold for up to 48 days. The findings agree with Azabou et al. [3], who showed that ROO that included tomato by-product extract of 500 mg kg^−1^ was lower in conjugated dienes and conjugated trienes compared to the control sample.

### 3.5. LSSCE and Chemical Kinetic of ROO Oxidation

**Figure 4:**
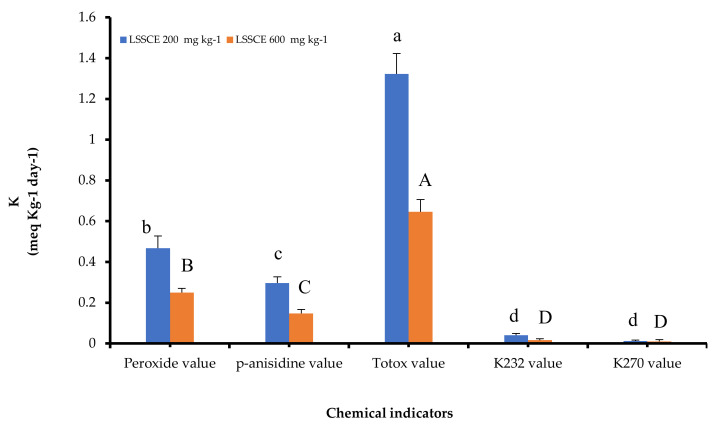
Impact of LSSCE 200 mg kg^−1^ and LSSCE 600 mg kg^−1^ on chemical indices, i.e., peroxide value, p-anisidine value, totox value, K_232_, and K_270_ of ROO. ^abcd^ no significant difference between any two means ʻin the blue columnʼ have the same superscript letter (*p* ≥ 0.05). ^ABCD^ no significant difference between any two means ʻin the orange columnʼ have the same superscript letter (*p* ≥ 0.05).

The chemical kinetic rat constants (k) of the key oil oxidation markers, such asthe peroxide value (PV), *p*-Anisidine value (AV), K_232_ value, and K_270_ value, and LSSCE at concentrations of 200 and 600 mg kg^−1^ were evaluated (Figure 4). A significant difference was observed in oxidation indicators between ROO samples containing LSSCE at 200 and 600 mg kg^−1^. The k value of oxidation indicators at 600 mg kg^−1^ were lower than at 200 mg kg^−1^. In ROO samples containing LSSCE 600 mg kg^−1^, the k values of PV, AV, K_232_, and K_270_ were 0.250 meq kg^−1^, 0.147 mg kg^−1^, 0.016 (at 232 nm), and 0.010 (at 270 nm), respectively. However, in ROO samples with LSSCE 200 mg kg^−1^, the k values were 0.468 meq kg^−1^, 0.297 mg kg^−1^, 0.041 (at 232 nm), and 0.012 (at 270 nm), respectively. A lower k value signifies that lipid rancidity is occurring at a slower rate. The results confirmed that sesame coats improved the oxidation resistance at high concentrations [14].

### 3.6. Antioxidant Remarks

In Table 3, the antioxidant levels of the ROO treatments were evaluated. The results revealed that all ROO treated were notably higher in the induction period (IP) than the control group. The IP values in ROO treatments included an LSSCE between 17.76 to 23.17 h. A significant difference in IP value was noted of ROO incorporating various levels of LSSCE (*p* ≤ 0.05). Although the IP value of samples containing LSSCE 600 mg kg^−1^ (23.17 h) was higher than that of 200 mg kg^−1^ (17.76 h), BHT treatment exhibited the highest IP (27.15 h). As shown in Table 3, there was no difference in the antioxidant activity (AA) among LSSCE treatments. However, remarkable differences were displayed in AA among LSSCE and BHT treatments. The antioxidant power (AOP) value of ROO included an LSSCE ranging from 10.98 to 31.77%. A high level of LSSCE (600 mg kg^−1^) in ROO exhibited outstandingly active amounts of AOP. The ROO with BHT had the highest AOP value of all, followed by LSSCE 600 mg kg^−1^ and LSSCE 400 mg kg^−1^. The improved oxidative stability (IOS) value of ROO treatments incorporating 200, 400, and 600 mg kg^−1^ of LSSCE were 12.33, 27.96, and 46.55%, respectively (Table 3). In general, LSSCE addition in ROO notably increased the IOS value. These findings are consistent with Morsy et al. [28], who found that spirulina extract improved antioxidant power (AOP) and oxidative stability (ISO) and extended the induction period (IP) in ROO.

### 3.7. Organoleptic Assessment of ROO

The organoleptic assessment of ROO treatments that was performed at various stages of storage until end of shelf life with off-flavor Table 4. In general, all treatments are free from any major changes, such as musty, bitter, and vinegary properties, during storage. The color score declined in mostly ROO treatments with progress storage; however, no notable difference (*p* ≥ 0.05) was noted up to day 48 between BHT and LSSCE treatments. On the other hand, the ROO control group exhibited that the lowest color value LSSCE addition at various concentrations had a favorable effect on the ROO color. Significant differences (*p* ≤ 0.05) were observed in the aroma value between ROO samples and the control. The aroma in ROO included antioxidants, i.e., BHT or LSSCE, was desirable until 48 days of storage, while the control sample was rejected on day 24. The acceptability scores of samples decreased mostly with storage time in treated ROO, while no notable difference was noted up to 48 days between BHT and LSSCE samples. ROO samples remained relatively stable for 18 days (control), with oxidation visible in the treated ROO depending on storage period: on day 30 (LSSCE 200 mg kg^−1^), on day 36 (LSSCE 400 mg kg^−1^), and on day 48 (LSSCE 600 mg kg^−1^ and BHT). 

**Figure 5:**
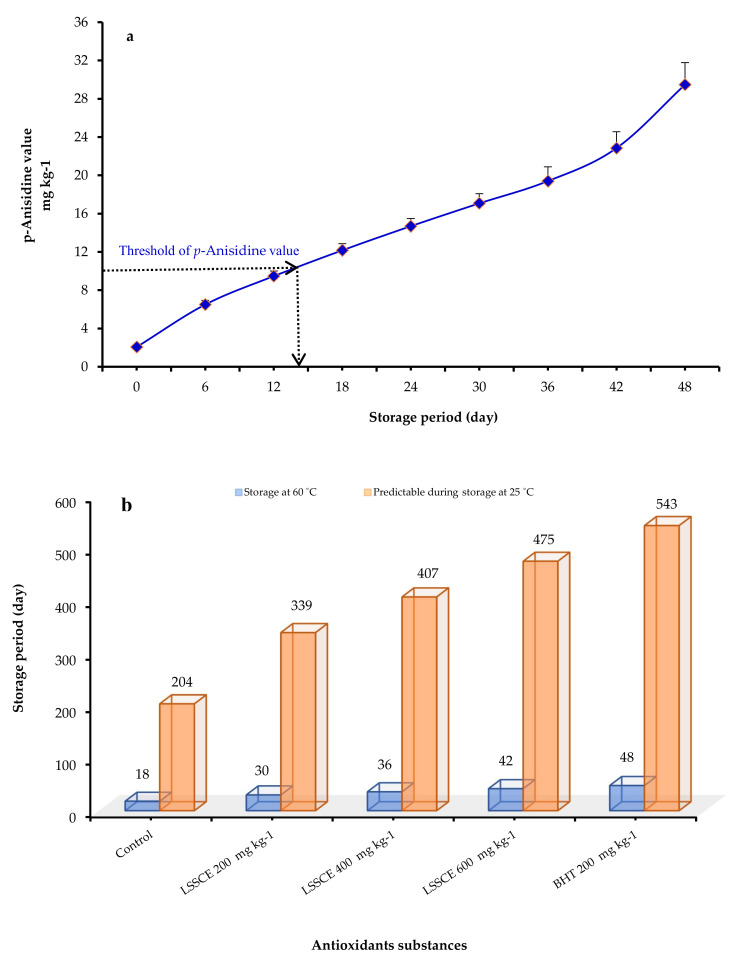
(**a**) Threshold of the *p*-anisidine value when ROO is stored at 60 °C up to 48 days. (**b**) The shelf life of ROO stored at 60 °C and the predicted shelf life at ambient temperature (25 °C).

Figure 5a demonstrates the assessment of ROO’s chemical indicators and organoleptic properties. The ROO control group was rejected at day 18 and was regarded as rancid on day 14 due to the *p*-anisidine value (threshold/limit of *p*-anisidine value10 mg kg^−1^). The *p*-anisidine increases extraordinarily from day 12–18 when the intensity of the sensory response reaches a climax, referring to a possibility of refusal. These findings corroborate those reported by Morsy et al. [28].

Based on the data provided by oxidation markers, sensory evaluation, and assuming Q_10_, the shelf life of ROO samples kept at 60 °C and the predicted shelf life at ambient conditions (25 °C) were plotted in (Figure 5b). The data illustrated that the shelf life of ROO under accelerated storage conditions (60 °C) was 18 days for the control, 30 days for 200 mg kg^−1^ LSSCE, 36 days for 400 mg kg^−1^ LSSCE, 42 days for 600 mg kg^−1^ LSSCE, and 48 days for BHT. At 25 °C, the predicted shelf life of ROO treatments was 204, 339, 407, 475, and 543 days, respectively. These figures were derived by assuming a Q10 value of 2.0 for oil oxidation. Thus, the acceleration factor was approximately 11.31 days, which means that one day at accelerated conditions (60 °C) is equivalent to 11.31 days at ambient conditions (25 °C) [62].

## 4. Conclusions

In conclusion, the lyophilized sesame seed coats (LSSCE)have been tested, among others, in terms of the content of phenols and flavonoids, antioxidant capacity, fatty acid profile, determination of olive oil quality parameters, and chemical kinetics. The authors showed that LSSCEs are a promising source of bioactive compounds, such as phenolic compounds and lignins based on HPLC/DAD. The LSSCE at different levels (200, 400, and 600 mg kg^−1^) demonstrated an effective antioxidant impact. LSSCE enhanced the stability of ROO-treated samples through the storage period compared with the control. The sample containing LSSCE showed significantly lowervalues of peroxide, *p*-anisidine, K_232_, and K_270_ than the control sample. The chemical kinetics (k) value was low in ROO samples, including BHT and LSSCE 600 mg kg^−1^, compared with other samples. The antioxidant treatment and organoleptic results of the ROO containing LSSCE were significantly higher than that of the control group. The results show that LSSCE improves the stability, quality, acceptability, and durability of ROO by up to several days at 25 °C.

## Figures and Tables

**Table 1:** Fatty acids composition (%) of refined olive oil (ROO).

Fatty Acids	ROO
Palmitic acid (C 16:0)	12.32 ± 0.07
Palmitoleic acid (C 16:1)	0.38 ± 0.03
Margaric acid (C 17:0)	0.05 ± 0.01
Margaroleic (C 17:1)	0.04 ± 0.01
Stearic acid (C 18:0)	3.12 ± 0.04
Oleic acid (C 18:1)	73.35 ± 0.01
Linoleic acid (C 18:2)	9.12 ± 0.14
α-Linolenic acid (C 18:3n-3)	0.61 ± 0.05
Arachidic acid (C 20:0)	0.6 ± 0.01
Eicosenoic acid (C 20:1)	0.41 ± 0.02
Saturated fatty acid ΣSFA	16.09 ± 0. 23
Monounsaturated fatty acid ΣMUFA	74.18 ± 0.22
Polyunsaturated fatty acid ΣPUFA	9.73 ± 0.12

ND, non-detectable, defined as ≤0.05%; SFA, saturated fatty acid (%); MUFA, monounsaturated fatty acid (%); PUFA, polyunsaturated fatty acid (%).

**Table 2:** Total phenolic (TP) and total flavonoid (TF) contents and radical scavenging activity of lyophilized sesame seed coats extract (LSSCE).

Sample	TP (mg g^−1^ dw)	TF (mg g^−1^ dw)	IC_50_ (μg mL^−1^)
Sesame seed coats (SSCs)	85.12 ± 1.33 ^b^	7.45 ± 0.23 ^b^	15.64 ± 0.96 ^a^
Lyophilized sesame seed coats extract (LSSCE)	105.9 ± 2.98 ^a^	9.00 ± 0.41 ^a^	9.90 ± 0.65 ^c^
α-tocopherol	“—”	“—”	14.28 ± 0.82 ^b^
BHT	“—”	“—”	5.05 ± 0.87 ^d^

Values in the same column with different letters are significantly different at *p* < 0.05.

**Table 3:** Impact of different levels of LSSCE on antioxidant indices of ROO.

Samples	IP (h)	AOP (%)	IOS (%)
Control	15.81 ± 0.23 ^e^	-	-
LSSCE 200 mg kg^−1^	17.76 ± 0.42 ^b^	10.98 ± 0.22 ^d^	12.33 ± 0.25 ^b^
LSSCE 400 mg kg^−1^	20.23 ± 0.50 ^c^	21.85 ± 0.48 ^c^	27.96 ± 0.38 ^c^
LSSCE 600 mg kg^−1^	23.17 ± 0.35 ^b^	31.77 ± 0.66 ^b^	46.55 ± 0.52 ^b^
BHT 200 mg kg^−1^	27.15 ± 0.6 ^a^	41.77 ± 0.53 ^a^	71.73 ± 0.85 ^a^

Values in the same column with different letters are significantly different at *p* < 0.05. IP: Induction period, AOP: antioxidant power, IOS: Improved oxidative stability.

**Table 4:** Organoleptic responses for color, aroma, and acceptability of refined olive oil (ROO) samples stored at 60 °C.

Attributes	Treatments	Storage Days								
		0	6	12	18	24	30	36	42	48
**Color**	Control	7.00 ± 0.00 ^aA^	5.20 ± 0.82 ^bB^	3.37 ± 0.40 ^cC^	2.16 ± 0.33 ^dC^	*R	R	R	R	R
	LSSCE 200 mg kg^−1^	7.00 ± 0.00 ^aA^	5.90 ± 0.32 ^bB^	5.00 ± 0.22 ^cB^	4.23 ± 0.47 ^dB^	3.40 ± 0.33 ^eC^	2.57 ± 0.18 ^fC^	R	R	R
	LSSCE 400 mg kg^−1^	7.00 ± 0.00 ^aA^	6.25 ± 0.63 ^aA^	5.83 ± 0.38 ^bA^	5.11 ± 0.32 ^bB^	4.85 ± 0.42 ^cB^	4.40 ± 0.56 ^cB^	3.22 ± 0.31 ^dB^	R	R
	LSSCE 600 mg kg^−1^	7.00 ± 0.00 ^aA^	6.60 ± 0.30 ^aA^	6.00 ± 0.44 ^bA^	5.77 ± 0.54 ^bA^	5.47 ± 0.32 ^bA^	5.15 ± 0.43 ^bA^	4.88 ± 0.23 ^cA^	4.13 ± 0.12 ^cA^	3.70 ± 0.22 ^dA^
	BHT 200 mg kg^−1^	7.00 ± 0.00 ^aA^	6.87 ± 0.55 ^aA^	6.28 ± 0.37 ^aA^	5.94 ± 0.53 ^bA^	5.65 ± 0.40 ^bA^	5.36 ± 0.33 ^bA^	4.95 ± 0.47 ^cA^	4.58 ± 0.26 ^cA^	3.95 ± 0.35 ^dA^
**Aroma**	Control	7.00 ± 0.00 ^aA^	6.90 ± 0.11 ^aA^	4.75 ± 0.44 ^bB^	2.10 ± 0.33 ^cC^	R	R	R	R	R
	LSSCE 200 mg kg^−1^	7.00 ± 0.00 ^aA^	7.00 ± 0.00 ^aA^	6.25 ± 0.18 ^aA^	4.50 ± 0.38 ^bB^	3.15 ± 0.34 ^cC^	2.40 ± 0.23 ^cC^	R	R	R
	LSSCE 400 mg kg^−1^	7.00 ± 0.00 ^aA^	7.00 ± 0.00 ^aA^	6.86 ± 0.00 ^aA^	6.45 ± 0.40 ^aA^	5.90 ± 0.57 ^bB^	4.40 ± 0.41 ^cB^	3.55 ± 0.27 ^dB^	R	R
	LSSCE 600 mg kg^−1^	7.00 ± 0.00 ^aA^	7.00 ± 0.00 ^aA^	7.00 ± 0.00 ^aA^	7.00 ± 0.00 ^aA^	6.90 ± 0.45 ^aA^	6.73 ± 0.33 ^aA^	6.20 ± 0.24 ^aA^	5.66 ± 0.37 ^bA^	4.58 ± 0.45 ^cA^
	BHT 200 mg kg^−1^	7.00 ± 0.00 ^aA^	7.00 ± 0.00 ^aA^	7.00 ± 0.00 ^aA^	7.00 ± 0.00 ^aA^	7.00 ± 0.00 ^aA^	7.00 ± 0.00 ^aA^	6.92 ± 0.42 ^aA^	6.45 ± 0.30 ^aA^	5.10 ± 0.24 ^bA^
**Acceptability**	Control	7.00 ± 0.00 ^aA^	6.85 ± 0.13 ^aA^	4.56 ± 0.33 ^bC^	3.12 ± 0.26 ^cC^	R*	R	R	R	R
	LSSCE 200 mg kg^−1^	7.00 ± 0.00 ^aA^	7.00 ± 0.00 ^aA^	6.15 ± 0.25 ^bB^	5.73 ± 0.41 ^bB^	4.22 ± 0.25 ^cC^	3.23 ± 0.15 ^dC^	R	R	R
	LSSCE 400 mg kg^−1^	7.00 ± 0.00 ^aA^	7.00 ± 0.00 ^aA^	6.45 ± 0.32 ^aA^	5.86 ± 0.23 ^bB^	5.12 ± 0.34 ^bB^	4.85 ± 0.37 ^cB^	3.42 ± 0.18 ^dB^	R	R
	LSSCE 600 mg kg^−1^	7.00 ± 0.00 ^aA^	7.00 ± 0.00 ^aA^	7.00 ± 0.00 ^aA^	6.90 ± 0.22 ^aA^	6.35 ± 0.38 ^aA^	5.95 ± 0.23 ^bA^	5.32 ± 0.32 ^bA^	4.93 ± 0.35 ^cA^	3.84 ± 0.12 ^dA^
	BHT 200 mg kg^−1^	7.00 ± 0.00 ^aA^	7.00 ± 0.00 ^aA^	7.00 ± 0.00 ^aA^	7.00 ± 0.00 ^aA^	6.92 ± 0.25 ^aA^	6.65 ± 0.30 ^aA^	5.74 ± 0.22 ^bA^	5.25 ± 0.16 ^bA^	4.36 ± 0.25 ^cA^

^abc^ Values in the same row with different letters are significantly different at *p* < 0.05. ^ABC^ Values in the same column with different letters are significantly different at *p* < 0.05., *R: it is mean the sample was rejected by panelists due to unpleasant color or aroma or acceptability. *n* = 10.

## Data Availability

Data is contained within the article.

## References

[1] Durante de Oliveira S., Araújo C.M., da Silva Campelo Borges G., dos Santos Lima M., Viera V.B., Garcia E.F., de Souza E.L., de Oliveira M.E.G. (2020). Improvement in physicochemical characteristics, bioactive compounds and antioxidant activity of acerola (*Malpighia emarginata* DC) and guava (*Psidium guajava* L.) fruit by-products fermented with potentially probiotic lactobacilli. LWT.

[2] Monteiro G.C., Minatel I.O., Pimentel A., Gomez-Gomez H.A., de Camargo J.P.C., Diamante M.S., Basílio L.S.P., Tecchio M.A., Lima G.P.P. (2021). Bioactive compounds and antioxidant capacity of grape pomace flours. LWT.

[3] Azabou S., Sebii H., Taheur F.B., Abid Y., Jridi M., Nasri M. (2020). Phytochemical profile and antioxidant properties of tomato by-products as affected by extraction solvents and potential application in refined olive oils. Food Biosci..

[4] Silva Y.P.A., Borba B.C., Pereira V.A., Reis M.G., Caliari M., Brooks M.S.-L., Ferreira T.A. (2019). Characterization of tomato processing by-product for use as a potential functional food ingredient: Nutritional composition, antioxidant activity and bioactive compounds. Int. J. Food Sci. Nutr..

[5] Fasihnia S.H., Peighambardoust S.H., Peighambardoust S.J., Oromiehie A., Soltanzadeh M., Peressini D. (2020). Migration analysis, antioxidant, and mechanical characterization of polypropylene-based active food packaging films loaded with BHA, BHT, and TBHQ. J. Food Sci..

[6] Mishra S.K., Belur P.D., Iyyaswami R. (2021). Use of antioxidants for enhancing oxidative stability of bulk edible oils: A review. Int. J. Food Sci. Technol..

[7] Calcio Gaudino E., Colletti A., Grillo G., Tabasso S., Cravotto G. (2020). Emerging processing technologies for the recovery of valuable bioactive compounds from potato peels. Foods.

[8] Morsy M.K., Mekawi E., Elsabagh R. (2018). Impact of pomegranate peel nanoparticles on quality attributes of meatballs during refrigerated storage. LWT.

[9] Felice F., Fabiano A., De Leo M., Piras A.M., Beconcini D., Cesare M.M., Braca A., Zambito Y., Di Stefano R. (2020). Antioxidant effect of cocoa by-product and cherry polyphenol extracts: A comparative study. Antioxidants.

[10] Centrone M., D’Agostino M., Difonzo G., De Bruno A., Di Mise A., Ranieri M., Montemurro C., Valenti G., Poiana M., Caponio F. (2021). Antioxidant Efficacy of Olive By-Product Extracts in Human Colon HCT8 Cells. Foods.

[11] Mujtaba M., Cho H.M., Masjuki H., Kalam M., Ong H., Gul M., Harith M., Yusoff M. (2020). Critical review on sesame seed oil and its methyl ester on cold flow and oxidation stability. Energy Rep..

[12] Hamza M., Abd El-Salam R. (2015). Optimum planting date for three sesame cultivars growing under sandy soil conditions in Egypt. Am.-Eurasian J. Agric. Environ. Sci..

[13] FAOSTAT (2017). Food and Agriculture Organization Statistics; Production Yearbook. http://www.fao.org/faostat/en/#data/QD/visualize.

[14] El-Roby A.M., Hammad K.S.M., Galal S.M. (2020). Enhancing oxidative stability of sunflower oil with sesame (*Sesamum indicum*) coat ultrasonic extract rich in polyphenols. J. Food Processing Preserv..

[15] Catargiu A.D., Raican D.-D., Poiana M.-A. (2017). Innovative approaches to improve the quality attributes of halva: A review. J. Agroaliment. Processes Technol..

[16] Elleuch M., Besbes S., Roiseux O., Blecker C., Attia H. (2007). Quality characteristics of sesame seeds and by-products. Food Chem..

[17] Zouari R., Besbes S., Ellouze-Chaabouni S., Ghribi-Aydi D. (2016). Cookies from composite wheat–sesame peels flours: Dough quality and effect of Bacillus subtilis SPB1 biosurfactant addition. Food Chem..

[18] Elleuch M., Bedigian D., Besbes S., Blecker C., Attia H. (2012). Dietary fibre characteristics and antioxidant activity of sesame seed coats (testae). Int. J. Food Prop..

[19] Wang B.-S., Chang L.-W., Yen W.-J., Duh P.-D. (2007). Antioxidative effect of sesame coat on LDL oxidation and oxidative stress in macrophages. Food Chem..

[20] Ortega-Hernández E., Coello-Oliemans C., Ornelas-Cravioto A., Santacruz A., Becerra-Moreno A., Jacobo-Velázquez D.A. (2018). Phytochemical characterization of sesame bran: An unexploited by-product rich in bioactive compounds. CyTA-J. Food.

[21] Mehdi L., Ahmed Z., Ouassila L., Abdeslam-Hassen M. Supereritical fluid extraction of oil from (de-hulled and ground) sesame seeds: Statistical and phenomenological modelling. Proceedings of the 2020 11th International Renewable Energy Congress (IREC).

[22] Prakash K., Naik S.N., Yadav U. (2020). Effects of Sesame Seed Oil (Black/White) as a Natural Antioxidant on the Oxidative and Frying Stability of Linseed Oil. Eur. J. Nutr. Food Saf..

[23] ES (2005). Egyptian Standards. Vegetable Edible Oils Part 2-No. 49, Olive Oils and Olive Pomace Oils.

[24] FAO, WHO (2013). Standard for Olive Oils and Olive Pomace Oils.

[25] Medeiros D.M., Hampton M. (2019). Olive oil and health benefits. Handbook of Nutraceuticals and Functional Foods.

[26] Haldar S., Wong L.H., Tay S.L., Jacoby J.J., He P., Osman F., Ponnalagu S., Jiang Y.R., Lian H.P.R., Henry C.J. (2020). Two Blends of Refined Rice Bran, Flaxseed, and Sesame Seed Oils Affect the Blood Lipid Profile of Chinese Adults with Borderline Hypercholesterolemia to a Similar Extent as Refined Olive Oil. J. Nutr..

[27] Kehili M., Choura S., Zammel A., Allouche N., Sayadi S. (2018). Oxidative stability of refined olive and sunflower oils supplemented with lycopene-rich oleoresin from tomato peels industrial by-product, during accelerated shelf-life storage. Food Chem..

[28] Morsy M.K., Morsy O.M., Elbarbary H.A., Saad M.A. (2019). Enhancing of oxidative stability and quality attributes of olive oil using spirulina (*Arthrospira platensis*) nanoparticles. LWT.

[29] Kehili M., Sayadi S., Frikha F., Zammel A., Allouche N. (2019). Optimization of lycopene extraction from tomato peels industrial by-product using maceration in refined olive oil. Food Bioprod. Processing.

[30] Hammouda I.B., Márquez-Ruiz G., Holgado F., Sonda A., Skalicka-Wozniak K., Bouaziz M. (2020). RP-UHPLC–DAD-QTOF-MS As a Powerful Tool of Oleuropein and Ligstroside Characterization in Olive-Leaf Extract and Their Contribution to the Improved Performance of Refined Olive-Pomace Oil during Heating. J. Agric. Food Chem..

[31] Tabaraki R., Heidarizadi E., Benvidi A. (2012). Optimization of ultrasonic-assisted extraction of pomegranate (*Punica granatum* L.) peel antioxidants by response surface methodology. Sep. Purif. Technol..

[32] Hajimahmoodi M., Faramarzi M.A., Mohammadi N., Soltani N., Oveisi M.R., Nafissi-Varcheh N. (2010). Evaluation of antioxidant properties and total phenolic contents of some strains of microalgae. J. Appl. Phycol..

[33] Formagio A.S.N., Volobuff C.R.F., Santiago M., Cardoso C.A.L., Vieira M.D.C., Valdevina Pereira Z. (2014). Evaluation of antioxidant activity, total flavonoids, tannins and phenolic compounds in Psychotria leaf extracts. Antioxidants.

[34] Liu X., Ardo S., Bunning M., Parry J., Zhou K., Stushnoff C., Stoniker F., Yu L., Kendall P. (2007). Total phenolic content and DPPH radical scavenging activity of lettuce (*Lactuca sativa* L.) grown in Colorado. LWT-Food Sci. Technol..

[35] Ebrahimzadeh M.A., Nabavi S.M., Nabavi S.F., Bahramian F., Bekhradnia A.R. (2010). Antioxidant and free radical scavenging activity of *H. officinalis L. var. angustifolius*, *V. odorata*, *B. hyrcana* and *C. speciosum*. Pak. J. Pharm. Sci..

[36] Kim K.W., Thomas R. (2007). Antioxidative activity of chitosans with varying molecular weights. Food Chem..

[37] Chang L.-W., Yen W.-J., Huang S.C., Duh P.-D. (2002). Antioxidant activity of sesame coat. Food Chem..

[38] Sukumar D., Arimboor R., Arumughan C. (2008). HPTLC fingerprinting and quantification of lignans as markers in sesame oil and its polyherbal formulations. J. Pharm. Biomed. Anal..

[39] Ackman R.G. (2002). The gas chromatograph in practical analyses of common and uncommon fatty acids for the 21st century. Anal. Chim. Acta.

[40] AOCS (2009). Official Methods and Recommended Practices of the AOCS.

[41] Paquot C., Hautfenne A. (1987). Standard Methods for the Analysis of Oils, Fats, and Derivatives.

[42] Gómez-Alonso S., Mancebo-Campos V., Desamparados Salvador M., Fregapane G. (2004). Oxidation kinetics in olive oil triacylglycerols under accelerated shelf-life testing (25–75 °C). Eur. J. Lipid Sci. Technol..

[43] Steele R. (2004). Understanding and Measuring the Shelf-Life of Food.

[44] Symoniuk E., Ratusz K., Ostrowska-Ligęza E., Krygier K. (2018). Impact of selected chemical characteristics of cold-pressed oils on their oxidative stability determined using the rancimat and pressure differential scanning calorimetry method. Food Anal. Methods.

[45] International Olive Oil Council (2015). Trade Standard Applying to Olive Oil and Olive-Pomace Oil.

[46] International Olive Oil Council (2010). Sensory Analysis of Olive Oil Method for the Organoleptic Assessment of Virgin Olive Oil.

[47] Bongartz A., Oberg D. (2011). Sensory evaluation of Extra Virgin Olive Oil (EVOO) extended to include the quality factor “harmony”. J. Agric. Sci. Technol..

[48] Steel R., Torrie J. (1980). Principles and Procedures of Statistics: A Biometrical Approach.

[49] Alavi N., Golmakani M.-T. (2017). Improving oxidative stability of virgin olive oil by addition of microalga Chlorella vulgaris biomass. J. Food Sci. Technol..

[50] Saad A.B., Jerbi A., Khlif I., Ayedi M., Allouche N. (2020). Stabilization of Refined Olive Oil with Phenolic Monomers Fraction and Purified Hydroxytyrosol from Olive Mill Wastewater. Chem. Afr..

[51] Genovese A., Caporaso N., Villani V., Paduano A., Sacchi R. (2015). Olive oil phenolic compounds affect the release of aroma compounds. Food Chem..

[52] Shahidi F., Liyana-Pathirana C.M., Wall D.S. (2006). Antioxidant activity of white and black sesame seeds and their hull fractions. Food Chem..

[53] Sarkis J.R., Michel I., Tessaro I.C., Marczak L.D.F. (2014). Optimization of phenolics extraction from sesame seed cake. Sep. Purif. Technol..

[54] Elhanafi L., Benkhadda Z.B., Rais C., Houhou M., Lebtar S., Channo A., Greche H. (2020). Biochemical Composition, Antioxidant Power and Antiinflammatory of Dehulled Sesamum indicum Seeds and Its Coat Fraction. Jordan J. Biol. Sci..

[55] Lin X., Zhou L., Li T., Brennan C., Fu X., Liu R.H. (2017). Phenolic content, antioxidant and antiproliferative activities of six varieties of white sesame seeds (*Sesamum indicum* L.). Rsc. Adv..

[56] Wang L., Zhang Y., Li P., Wang X., Zhang W., Wei W., Zhang X. (2012). HPLC analysis of seed sesamin and sesamolin variation in a sesame germplasm collection in China. J. Am. Oil Chem. Soc..

[57] Shi L.K., Liu R.J., Jin Q.Z., Wang X.G. (2017). The contents of lignans in sesame seeds and commercial sesame oils of China. J. Am. Oil Chem. Soc..

[58] Chong Y.M., Chang S.K., Sia W.C.M., Yim H.S. (2015). Antioxidant efficacy of mangosteen (*Garcinia mangostana* Linn.) peel extracts in sunflower oil during accelerated storage. Food Biosci..

[59] Frankel E. (2014). Lipid Oxidation.

[60] Tabee E., Jägerstad M., Dutta P.C. (2009). Frying quality characteristics of French fries prepared in refined olive oil and palm olein. J. Am. Oil Chem. Soc..

[61] Zhou B., Sun Y., Li J., Long Q., Zhong H. (2018). Effects of Seed Coat on Oxidative Stability and Antioxidant Activity of Apricot (*Prunus armeniaca* L.) Kernel Oil at Different Roasting Temperatures. J. Am. Oil Chem. Soc..

[62] Gharby S., Harhar H., Mamouni R., Matthäus B., Charrouf Z. (2016). Chemical Characterization and Kinetic parameter determination under Rancimat test conditions of four monovarietal virgin olive oils grown in Morocco. OCL.

